# Patterns of Tyrosine Kinase Inhibitor Utilization in Newly Treated Patients With Chronic Myeloid Leukemia: An Exhaustive Population-Based Study in France

**DOI:** 10.3389/fonc.2021.675609

**Published:** 2021-09-30

**Authors:** Marie Pajiep, Cécile Conte, Françoise Huguet, Martin Gauthier, Fabien Despas, Maryse Lapeyre-Mestre

**Affiliations:** ^1^ Service de Pharmacologie Médicale et Clinique, Centre Hospitalier Universitaire de Toulouse, Toulouse, France; ^2^ Equipe PEPSS (Pharmacologie en Population, cohorteS, biobanqueS), Centre d’Investigation Clinique 1436, INSERM, Université de Toulouse 3, Toulouse, France; ^3^ Départment d’Hématologie, Institut Universitaire du Cancer de Toulouse, Centre Hospitalier Universitaire (CHU) de Toulouse, Toulouse, France

**Keywords:** chronic myeloid leukemia, incidence, polypharmacy, comorbidities, first line treatment, tyrosine kinase inhibitors (TKI)

## Abstract

We analyzed demographic characteristics, comorbidities and patterns of treatment with tyrosine kinase inhibitors (TKIs) in a cohort of 3,633 incident cases of chronic myeloid leukemia (CML) identified across France from 1 January 2011 to 31 December 2014. Patients were identified through a specific algorithm in the French Healthcare Data System and were followed up 12 months after inclusion in the cohort. The estimated incidence rate of CML for this period in France was 1.37 per 100,000 person-years (95% Confidence Interval 1.36-1.38) and was higher in men, with a peak at age 75-79 years. At baseline, the median age of the cohort was 60 years (Inter Quartile Range 47-71), the Male/Female ratio was 1.2, and 25% presented with another comorbidity. Imatinib was the first-line TKI for 77.6% of the patients, followed by nilotinib (18.3%) and dasatinib (4.1%). Twelve months after initiation, 86% of the patients remained on the same TKI, 13% switched to another TKI and 1% received subsequently three different TKIs. During the follow-up, 23% discontinued and 52% suspended the TKI. Patients received a mean of 16.7 (Standard Deviation (SD) 9.6) medications over the first year of follow-up, and a mean of 2.7 (SD 2.3) concomitant medications on the day of first TKI prescription: 24.4% of the patients received allopurinol, 6.4% proton pump inhibitors (PPI) and 6.5% antihypertensive agents. When treatment with TKI was initiated, incident CML patients presented with comorbidities and polypharmacy, which merits attention because of the persistent use of these concomitant drugs and the potential increased risk of drug-drug interactions.

## Introduction

Chronic myeloid leukemia (CML) is a myeloproliferative neoplasm characterized by the Philadelphia chromosome which is the source of a Bcr-Abl1 hybrid protein with constitutive tyrosine-kinase activity (1). Published data on the annual incidence of CML varies from as low as 0.4/100,000 persons in some non-Western countries to 1.75/100,000 in the USA (2–5). Reports from several European CML registries consistently show a crude annual incidence of 0.7–1.0/100,000 inhabitants, a median age at diagnosis of 57–60 years and a Male/Female (M/F) ratio of 1.2–1.7 (2, 6, 7). In France, some studies based on specific cancer registries for the period 1980-2009 estimate the CML incidence rate between 0.95 and 1.14 per 100,000 person-years with a mean age of 54,7 years and a M/F ratio of 1.22 to 1.36 (8, 9).

A more recent study based on a health insurance database estimated the prevalence of CML in France at 16.3 (95% Confidence Interval (95%CI): 16.0-16.6) per 100,000 inhabitants, with a median age of 63 years (Inter Quartile Range (IQR) 51-73) (10).

Since the 2000s, drugs specifically targeting the tyrosine-kinase activity of the BCR-ABL1 oncoprotein (Tyrosine Kinase Inhibitors: TKIs) have been on the market (11). The first to appear was imatinib, approved by the European Medicines Agency in November 2001 (12). Since then second (dasatinib in November 2006, nilotinib in November 2007, bosutinib in August 2013) and third generation (ponatinib in July 2013) TKIs have been approved. They were approved initially for imatinib-resistant or intolerant patients, and then dasatinib and nilotinib subsequently received approval as first-line treatments of chronic phase CML in 2010 (13). The prescription of TKI (all oral) is therefore systematic in any newly diagnosed case of CML (14, 15) and their advent has changed the prognosis of CML from hospital management in a life-threatening context to ambulatory management in patients whose average survival is now only slightly different from that of the general population (11, 15). Health insurance databases offer the opportunity to study real-life drug safety in general population (16–18). However, few studies describe the management of CML patients by TKI in the French general population since they have been available (19, 20). The SNDS (in French, “*Système National des Données de Santé”*, French Healthcare Data System; https://www.snds.gouv.fr/SNDS/) was created in the early 2000s, and includes healthcare data from the entire French population (≈66 million inhabitants) (21–24). Based on these exhaustive national data, the aim of the study was to characterize the patterns of TKI utilization among incident CML patients, with a focus on the type of TKI in first-line therapy. The secondary objectives were to describe comorbidities and comedications at the time of TKI initiation.

## Materials and Methods

### Data Source

The SNDS is an electronic healthcare database which centralizes the reimbursement data for over 98% of the French population (21, 24). Health insurance is mandatory in France with no exclusion according to professional activity or incomes. The SNDS contains individualized, anonymous comprehensive data on patient demographics, healthcare reimbursement and eligibility for full reimbursement of health care expenses related to long-term diseases (LTD) (in French “*Affections de Longue Durée*”). Available data include year of birth; gender; location; coverage by the CMU-c (in French “*Couverture Maladie Universelle*”, a complementary universal health coverage system for people with low incomes); vital status and date of death. It also include reimbursed outpatient healthcare expenditures such as medical visits, laboratory tests, drugs dispensed with the date and quantity supplied identified with the Anatomic Therapeutic and Chemical (ATC) classification; hospital discharge summaries including all diagnoses (main, related and up to 10 associated diagnoses) coded with the International Classification of Diseases, 10^th^ revision (ICD10) (25). There is a list of LTD, which includes 3448 ICD10 codes. Patients registered for these diseases benefit from full coverage for all medical expenses related to the disease for a period defined in the database. LTD registration is obtained at the request of a patient’s practitioner and validated by the health insurance system. The SNDS has been widely used to conduct large epidemiologic studies and further information regarding the organization has already been described elsewhere (26–28). Access to the exhaustive database is done under permissions dependent on the type of data requested, with a particular attention to avoid any re-identification. For this study, data available at the time of extraction covered the period 2010-2015.

### Study Design and Population Selection

We designed a retrospective national cohort study of all newly diagnosed patients for whom specific treatment was initiated with one of the TKIs approved in Europe for CML (imatinib, dasatinib, nilotinib, bosutinib and ponatinib). We selected all patients identified in the SNDS between 1 January 2010 and 31 December 2015 with at least one reimbursement for any of these TKIs and aged > 18 years old.

We used a specific algorithm to identify incident CML patients who were treated between 1 January 2011 (in order to have at least 12 months before TKI initiation for the identification of comorbidities) and 31 December 2014 (to have at least 12 months of follow-up for the last patients included, to investigate patterns of TKI utilization). The initial algorithm was previously used in a pilot study based on regional data extracted from the SNDS (27).

Patients were defined as incident CML patients with the following conditions:

- First reimbursement for a TKI between 01/01/2011 and 31/12/2014

AND

- First ICD10 codes for CML (C92.1 or C92) identified during a hospital stay or with LTD status between 01/01/2011 and 31/12/2014. Any mention of these codes before or after this period led to the patient’s exclusion. For a patient with an ICD10 code of CML (hospital or LTD) before the first reimbursement of TKI, the date of CML incidence was the date of appearance of the ICD10 code.

AND

- At least two dispensations of TKI

Patients could be treated with TKI not for a CML, but for another indication (either approved, or off-label). These diseases were identified through a list of different diagnosis codes and specific drugs presented in detail in **Supplementary File 1**. Patients with at least one of these other conditions were excluded.

In order to validate the suitability of this algorithm, we extracted a randomly selected sample of 20 electronic files reviewed independently by two hematologists. For each selected patient, reviewers verified the standardized patient form with demographic characteristics and sequences of care (12 months before and 12 months after the use of a TKI for each patient). Inter-rater concordance between the classification obtained by the algorithm and the opinion of the two reviewers was estimated and considered as good if ≥ 80%.

### Baseline Comorbidities and Care Consumption

Baseline comorbidities were those included in the Charlson’s comorbidities index (CCI), and registered with ICD10 diagnosis codes in the 12 months before index date. Consumption of care was described by the characterization of drugs, hospital stays and LTD conditions in the 12 months preceding inclusion.

The comorbidities were evaluated by the CCI constructed from the SNDS data. A previous study based on the application of this index in the SNDS has shown its validity in predicting one-year mortality for the French population (29). As all patients in the cohort had incident CML by definition, the CML entity in the cancer class was not included in the index calculation. Baseline comorbidities were presented as the aggregate comorbidities measure (CCI), categorized as 0, 1, 2 or more than 2 comorbidities, and as individual comorbidities included in the CCI.

Consumption of care in the 12 months preceding inclusion was described by the characterization of drugs (at least one reimbursement of drugs categorized with the ATC classification) and by the number of hospital stays and LTD conditions.

### Treatment Patterns During the First Year of Follow-Up

In order to describe treatment patterns we selected patients with at least two distinct dispensations of TKI within the first year of follow-up.

TKIs: Drugs of interest were exhaustively identified in the SNDS, as they are universally reimbursed. We described first-line TKI treatment (first TKI reimbursed) and classified them as first (imatinib), second (nilotinib, dasatinib, bosutinib) and third generation (ponatinib) (12). TKI sequences of treatment and switches between first and second generations within 12 months following the inclusion were also described.

Comedications: We described the number and distinct type of drugs prescribed to each patient (within 12 months after the inclusion).

### Statistical Analyses

The analyses were performed with SAS V9.4^®^ software. A descriptive analysis of the demographic and medical characteristics of the entire cohort was performed with the usual indicators: means and standard deviations (SDs) or medians and interquartile ranges (IQRs) depending on their normality, and absolute and relative frequencies. Eventually, patients who died after their inclusions were identified with their vital status and date of death (not cause of death, which was not available in the SNDS at the time of extraction). The overall survival probability was estimated through the mean of Kaplan Meier curves according to age and CCI. The overall cumulative incidence rate of newly treated CML patients and its 95%CI was estimated as a whole and by age group, gender and in the 22 main administrative regions of France.

For the cumulative incidence calculations, we use the following formula:


$$I_{c}=\frac{m[t,t+\Delta t]}{N_{0}[t,t+\Delta t]}\;\begin{array}{l} \text{m}=\text{number of incident cases during all the period}\\ {\text{N}}_{0}=\text{number of people not ill at the beginning of the period}\\ \Delta_{\text{t}}=\text{Time period} \end{array}$$

We used as denominator the French population data provided by the INSEE (in French “*Institut National de la Statistique et des Etudes Economiques*”, French Institute of Statistics and Economic Studies). A binary logistic regression was performed to assess relationship between interruption of treatment and switching with interruption as the dependent variable. In order to validate the descriptive results for the incident CML population that we constructed using our algorithm, we performed a sensitivity analysis by selecting only patients with an incident myeloid leukemia LTD code (C92) between 2011 and 2014.

### Ethical Requirements

All ethical authorizations were obtained (*Institut des Données de Santé* approval, no. 165, November 24, 2015; *Commission Nationale de l’Informatique et des Libertés* authorization, no. DE-2015-119, December 24, 2015). The data recorded in this study were processed in accordance with French Data Protection Act No. 78-17 of 6 January 1978, amended by Act No. 2004-801 of 6 August 2004. Final data were extracted and made available for analysis in January 2018.

## Results

### Population Selection and General Characteristics of the Cohort

**Figure 1:**
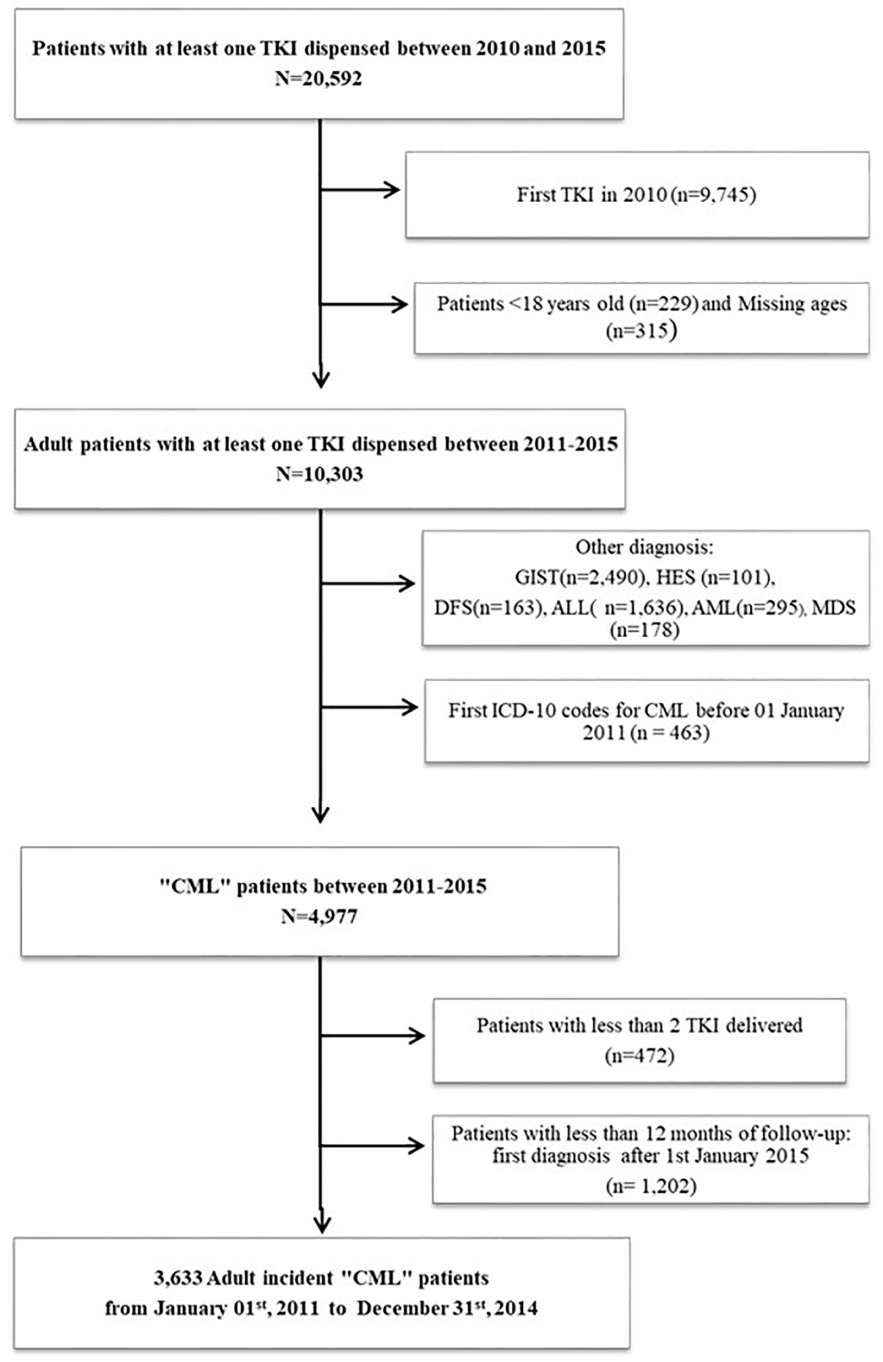
Flowchart illustrating patients’ selection. TKI, Tyrosin Kinase Inhibitor; CML, Chronic Myeloid Leukemia; GIST, Gastrointestinal Stromal Tumors; HES, Hypereosinophilic Syndromes; DFS, Dermatofibrosarcoma; ALL, Acute Lymphoblastic Leukemia; AML, Acute Myeloid Leukemia; MDS, Myelodysplastic Syndromes.

**Figure 2:**
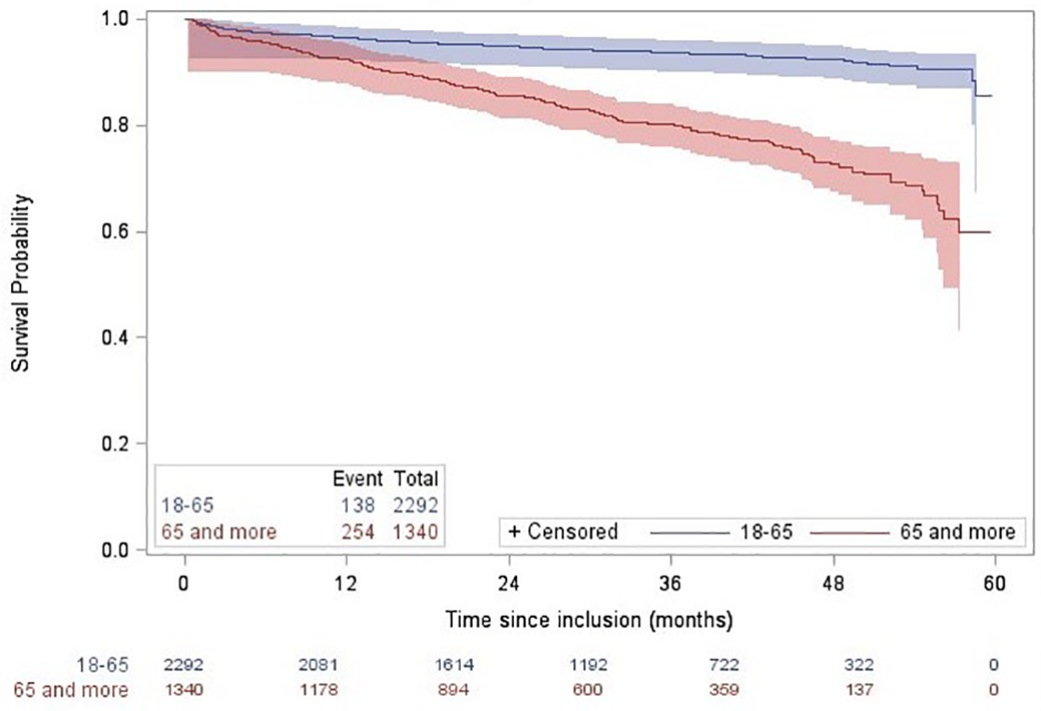
Overall survival probability estimated through Kaplan-Meier curves according to age classes in incident Chronic Myeloid Leukemia (CML) patients identified in the SNDS between 2011 and 2014 in France. The overall survival rate was significantly different between age groups, according to the Log rank test.

Between 1 January 2010 and 31 December 2015, 20,592 patients had at least one TKI reimbursement for CML. After the selection process (**Figure 1**), we identified a cohort of 3,633 patients who started TKI treatment and were diagnosed with CML between 1 January 2011 and 31 December 2014 in France. At the index date, patients had a median age of 60 years [IQR: 47-71], with 557 patients aged 18-39 years (15.3%), 1637 aged 40-64 (45.0%), 735 aged 65-74 (20.2%), and 704 patients over 75 years (19.4%). A majority was men (54.6%) with a M/F ratio of 1.2 **(** **Table 1** **).** The median duration of follow-up was 39 months (IQR: 27 – 48 months). At inclusion, only 1% (60) of patients was hospitalized for CML, increasing to 15% (536) during follow-up. We listed 2.6% (96 patients) deaths in the first year of follow-up, and overall 392 patients died during the study period, giving an overall survival probability of 89.21% (95%CI 88.20%-90.21%) at 5 years after inclusion (93.98% for patients 18-64 years old; 81.04% for patients over 65 years; p<0.05 according to the Log rank test) **(** **Figure 2** **).**

**Table 1:** Description of population characteristics and comorbidities (in the 12 months preceding the index date) in incident “CML” subjects identified in the SNDS, 2011-2014, France.

Characteristics	N (%)
**Number of subjects**	3,633
**Age, years - median (interquartile range)**	60 (47-71)
**Gender**	
** Men**	1,984 (54,6)
** Women**	1,649 (45,4)
**CMU-C status**	
** No**	3,363 (92,6)
** Yes**	221 (6,1)
**Comorbidities**	
**Subjects with at least one LTD in the 12months preceding the index date**	342 (9.4)
**Subjects with at least one hospitalization in the 12months preceding the index date**	1,251 (34.4)
**Charlson comorbidities index (CCI)**	
** 0**	2,738 (75.4)
** 1**	338 (9.3)
** 2**	305 (8.4)
** >2**	252 (6.9)
**Individual comorbidities according to CCI**	
** Cancer (without CML)**	402 (11.0)
** Chronic lung disease**	343 (9.4)
** Diabetes without complications**	313 (8.6)
** Metastatic pathology**	119 (3.6)
** Peripheral vascular disease**	95 (2.6)
** Moderate to severe renal disease**	87 (2.4)
** Diabetes with complications**	63 (1.7)
** Myocardial infarction**	61 (1.7)
** Heart failure**	56 (1.5)
** Cerebrovascular pathology**	47 (1.3)
** Dementia**	31 (0.8)
** Mild liver disease**	24 (0.7)
** Connectivity**	19 (0.5)
** Hemiplegia**	19 (0.5)
** Ulcerative pathology**	19 (0.5)
** Moderate to severe liver disease**	5 (0.1)
** HIV-AIDS**	6 (0.2)
**Drug classes**	
**Subjects with at least one drug prescribed in the 12months preceding the index date**	3,238 (89.1)
** Paracetamol**	2,253 (62.0)
** Proton pump inhibitors**	1,488 (40.9)
** Antihypertensive agents**	1,146 (31.5)
** NSAIDS**	1068 (29.3)
** Intestinal motility stimulants**	1,010 (27.8)
** Opioid analgesics**	977 (26.9)
** Benzodiazepines**	936 (25.7)
** Glucocorticoids**	956 (26.3)
** Antithrombotic**	868 (23.9)
** Statins**	772 (19.9)
** Vitamin D**	657 (18.1)
** Antihistaminic**	503 (13.8)
** Allopurinol**	402 (11.1)
** Antidepressants (SSRI)**	338 (9.3)
** Levothyroxine**	302 (8.3)

CMU-c, complementary universal healthcare coverage.

NSAIDs, Non-steroidal anti-inflammatory drugs; SSRI, Selective serotonin reuptake inhibitor.

### Algorithm Validation to Identify CML Patients

The comparison of the algorithm with the opinion of hematologists indicated an observed 90% concordance rate. According to the hematologists, the algorithm correctly identified 10 true CML patients and correctly excluded 8 false CML patients. The two remaining patients with discordant results were defined as having CML before 2011, and therefore were not considered as incident CML case.

### Incidence

**Figure 3:**
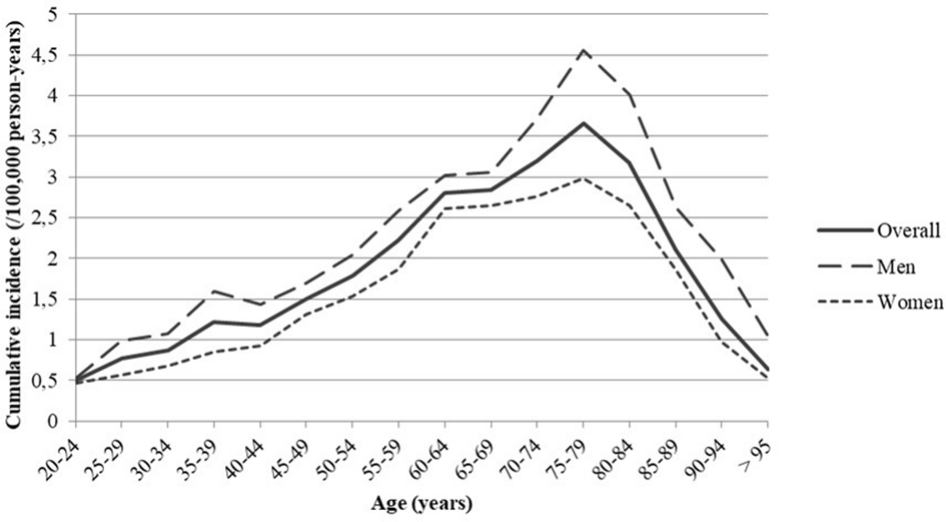
Cumulative incidence of Chronic Myeloid Leukemia (CML) in France during the period 2011-2014 by age and gender.

**Figure 4:**
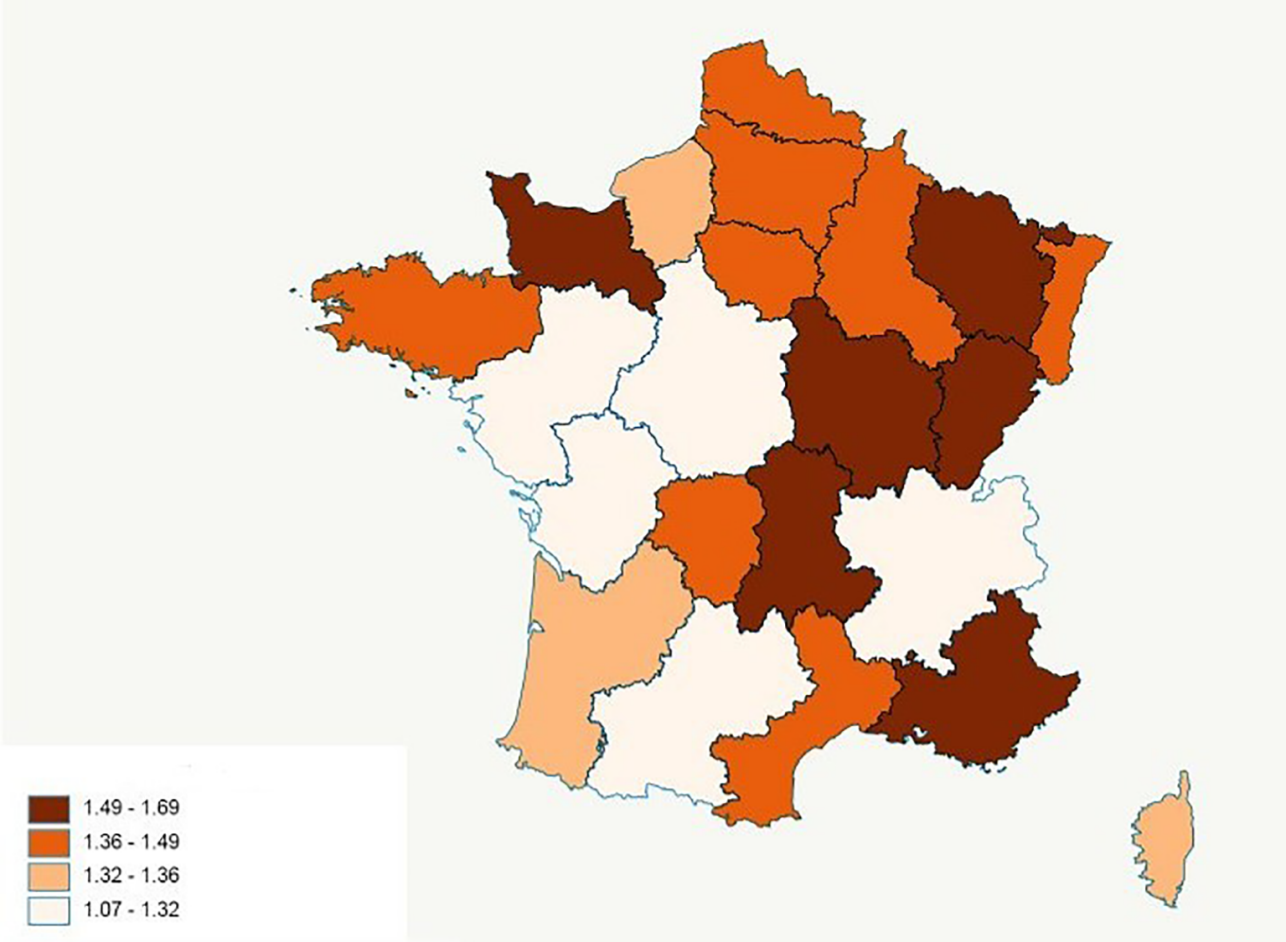
Distribution of the population of incident Chronic Myeloid Leukemia (CML) patient identified in the SNDS between 2011 and 2014 by regions of France. These are estimates of CML incidence per 100,000 inhabitants. Regions are those in which incident CML patients lived in 2014.

In our study, the cumulative incidence rate of CML over the period 2011-2014 was estimated at 1.37/100,000 person-years (95%CI 1.36-1.38). It was higher in men (1.54/100,000 person-years, 95%CI 1.53-1.55) than in women (1.20/100,000 person-years, 95%CI 1.19-1.21) and increased with age, reaching a peak at 75-79 years, after which it decreased. There was a male preponderance of CML in men in the different age groups **(** **Figure 3** **).** This incidence was relatively stable over each calendar year between 2011 and 2014 with an average of 908 (±25) incident cases per year. The cumulative incidence varied from 1.07 to 1.69 per 100,000 inhabitants across France. We observed differences in incidence between regions with a higher frequency trend in the east compared to the west of the country **(** **Figure 4** **).**

### Charlson’s Comorbidities Index and Care Consumption at Baseline

The **Table 1** presents the main characteristics of patients at baseline: 75.4% of the patients did not present another comorbidity included in the CCI at baseline, the CML being excluded. For patients with a CCI ≥ 1, the most frequently reported was other cancer (including lymphoid leukemia, tumors of uncertain prognosis, melanoma, etc.). Except for paracetamol which was taken at least once by 62.0% of the patients in the year before the index date, the main drugs used were proton pump inhibitors (PPI, 40.9% of patients), antihypertensive drugs (31.5%), non-steroidal anti-inflammatory drugs (NSAID, 29.3%), intestinal motility stimulants (27.8%) and opioid analgesics (26.9%) **(** **Table 1** **)**.

### TKI Patterns

Among the 3,633 patients in the cohort, imatinib was the most frequently delivered as the first-line treatment for 77.6% of the patients (n = 2,821), followed by nilotinib (18.3%, n = 663) and dasatinib (4.1%, n = 148). Only one patient was initiated with bosutinib (in 2013). In 2011, imatinib represented 81.6% of first line TKIs versus 11.4% for nilotinib, and 7.0% for dasatinib, whereas from 2012 to 2014, nilotinib represented 19% to 21%. Imatinib was used as the first line for 64.4% of patients 18-39 years old and for 90.5% of those over 75 years old. The majority of prescriptions corresponded to a treatment duration of 30 days (packs of 30 tablets delivered). The initial prescriber was mainly a hospital practitioner of the public sector (66%) and the median (IQR) time from first-line TKI to the end of follow-up was 36 months (26 – 47months).

In the year following the inclusion there were 3,480 patients with at least two distinct dispensations of TKI, on which we have performed the description of TKI and comedication patterns. Eighty-six percent of these patients received only one type of TKI during the first year of follow-up, 13% received two different TKIs and only 1% received subsequently 3 different TKIs. The median time (IQR) between two dispensations of TKI was 29 (26–34) days.

#### TKI Switch

68.5% of the patients (n = 2,384) were treated only by imatinib, 18.0% only had a second-generation TKI (with a predominance of nilotinib) and 13.6% received two different TKI (considered as a change in treatment during their care). Most of these switches (81%) occurred between imatinib and second-generation TKIs (either dasatinib or nilotinib). The median time (IQR) between the initiation of a CML TKI and a switch to another TKI was 5 (3-8) months. A higher proportion of patients (41%) had a TKI switch within 0-3 months after the start of treatment than in later periods.

#### TKI Interruption and Discontinuations

During this first year of follow-up, treatment discontinuations (defined as a cessation of TKI dispensation of more than 60 days before the end of following) were reported in 23% (n=818) of patients. Treatment interruptions (defined as temporary suspension of TKI dispensation, for more than 40 days) were observed in 52% (n=1,827) of the patients and approximately half of those patients (n=899) had more than one interruption. The median duration (IQR; min, max) of treatment interruption was 11 (4-22; 1, 319) days.

Of 473 patients who had a switch, 69% (n=328) also had an interruption of treatment and there was a significant relationship between switches and interruptions in treatment (Odds Ratio (OR) = 1.35; 95%CI 1.24-1.47). Most of the interruptions concerned patients with a TKI switch within 0-6 months after the start of treatment. Only 16% (n=76) of the patients who had a switch also had a discontinuation.

### Comedications

Throughout their care, patients were exposed to other drugs in addition to TKI. The mean number (SD) of concomitant medications at the start of TKI treatment was 2.7 (2.3) and 16.7 (9.6) over the first year of follow-up. The main drugs dispensed were paracetamol, PPIs, and antihypertensive drugs. Of the 3,480 patients, 51.3% (n=1,863) had at least one prescription of another drug on the day of the prescription for the first-line TKI and 71.5% (n=2,487) had a concomitant prescription within the month following the first TKI prescription **(** **Tables 2**, **3** **)**.

**Table 2:** Description of drug classes concomitant with TKI exposure (issued within the day of first TKI prescription) in the 3480 incident “CML” subjects with at least 2 dispensations of TKI, 2011-2014, France.

Drug classes	N (%)
** Allopurinol**	851 (24.4)
** Intestinal motility stimulants**	421 (12.1)
** Antihypertensive agents**	228 (6.5)
** Proton pump inhibitors**	223 (6.4)
** Antithrombotic agents**	211 (6.1)
** Paracetamol**	208 (6.0)
** NSAIDS**	185 (5.3)
** Other neoplastic**	155 (4.4)
** Benzodiazepines**	138 (3.9)
** Opioid analgesics**	135 (3.8)
** Statins**	75 (2.1)
** Glucocorticoids**	70 (2.0)
** Antiemetics**	66 (1.9)

NSAIDs, Non-steroidal anti-inflammatory drugs.

**Table 3:** Description of drug classes concomitant with TKI exposure (issued within the month of first TKI prescription) in the 3480 incident “CML” subjects with at least 2 dispensations of TKI, 2011-2014, France.

Drug classes	N (%)
** Antihypertensive agents**	815 (23.4)
** Proton pump inhibitors**	779 (22.4)
** Paracetamol**	689 (19.8)
** Allopurinol**	621 (17.8)
** Antithrombotic agents**	589 (16.9)
** Intestinal motility**	499 (14.3)
** Benzodiazepines**	487 (14.0)
** Statins**	387 (11.1)
** Opioid analgesics**	360 (10.3)
** NSAIDs**	298 (8.5)
** Thyroid hormones**	242 (7.0)
** Glucocorticoids**	198 (5.7)
** Antidepressants**	188 (5.4)
** Oral antidiabetics**	183 (5.2)
** Vitamin D**	175 (5.0)

NSAIDs, Non-steroidal anti-inflammatory drugs.

### Sensitivity Analysis

We selected patients who had at least one incident LTD record for myeloid leukemia (coded ICD-10 C92) between 1 January 2011 and 31 December 2014 from the 10,303 patients in the SNDS who had at least one incident reimbursement of TKI between 2011 and 2014. Three thousand one hundred and eighteen (3,118) patients were thereby identified, from whom all subjects with diagnostic codes for acute myeloid leukemia (AML: ICD-10 codes C920, C924, C925, C926 and C928) or acute lymphoblastic leukemia (ALL: ICD-10 code C910), either during hospitalization and/or in LTD, were excluded. With this method, we identified 2,812 patients who had an LTD with a potential incident “CML” between 01/01/2011 and 31/12/2014.

Characteristics of this population were similar to those of the CML population identified by the complete algorithm (**Supplementary File 2** **Table S1**).

## Discussion

### Main Results

The inclusion of 3,633 incident patients in 4 years makes this is one of the largest cohorts of incident patients with CML initially treated with TKI. This analysis of individual data from the French national health insurance databases provides original information on newly diagnosed CML patients in France at a nationwide scale. Median age at occurrence of CML was 60 years, and 52.2% of patients were 60 years or older with a M/F ratio of 1.2. Age and gender characteristics (aging population with increasing male preponderance) corroborate and update findings from previous studies on the incidence of CML in France (from a few cancer registries) and the prevalence at a regional and national level (8, 10). As reported in other studies, the overall survival rates in this population was similar to that of the general population (8, 30).

The incidence rate found in our study is within the range of population-based reports from Sweden, southeast England, the United Kingdom, Taiwan, and in a recently published study on CML patients in 20 European countries (**Table 4**). The incidence rates in these studies varied between 0.70 and 1.8/100,000 and the Surveillance, Epidemiology, and End Results (SEER) Program in the United States reported a remarkably high incidence of 1.75/100.000 (2, 3, 6, 7). Our incidence rate is higher than previous estimates of the incidence in France which are between 0.95 and 1.14 per 100,000 person-years **(** **Table 4** **)** (8, 9). These incidence rates were obtained from data of five to six French population-based cancer registries between 1980 and 2009. These registries cover approximately 8 million inhabitants in France, while we used individual data from the French national health insurance databases, which cover approximately 66 million inhabitants; we also covered a more recent period (2011-2014) than these studies. Therefore, the large size and aging of the French population in our study, may explain the increase in incidence.

**Table 4:** Chronic Myeloid Leukemia incidence and prevalence rates estimated from different population-based registries or surveys.

First author (year of publication) [ref]	Geographical area	Years	Prevalence rate (/100,000)	Incidence rate (/100,000)	Data source
**Chen et al. (Höglund et al.) (2013) (** 2 **)**	USA	1975–2009		1.75	US (SEERS): 17 tumor registries covering approximately 25% of the US population
**Gunnarsson (2016) (** 3 **)**	Sweden	1970-2012	3.9 in 1985 11.9 in 2012		Swedish Cancer Register and Swedish Cause of Death Register
**Delord et al. (2018) (** 9 **)**	France	1960 to 2060	2.5 before the 1980s, to 6 by 2002. 18 and 24 in 2018 and 2030.	0.95	Cohort component-based model using projections of the French population. Six cancer registries for incidence from 1980-2009
**Thielen N et al. (2015) (** 30 **)**	Netherlands	1989-2011 and 2001-2012		0.9 and 0.8	Nationwide population based Netherlands Cancer Registry (NCR)
**Visser et al. (2012) (** 28 **)**	Europe	1995-2002	5.6 in 2008	1.2	Europe (RARECARE project) : 22 European cancer registries
**Sant M et al. (2010) (** 6 **)**	Europe	2000-2002		1.10	Europe (HAEMACARE project) : 44 European cancer registries
**Penot et al. (2015) (** 8 **)**	France	1980-2009		1.14	Five cancer registries
**Höglund et al. (2015) (** 2 **)**	Sweden	2002–2010		0.9	Swedish Cancer Register
**Hoffman et al. (Höglund et al.) (2014) (** 2 **)**	Europe	2008-2012		0.7-1.0	European Treatment and Outcome. Study (EUTOS) for CML in 27 European countries
**Nguyen et al. (2018) (** 4 **)**	Canada	2011- 2015		0.87	Calgary Laboratory Services (CLS) Cancer Cytogenetics Laboratory
					
**Neves et al. (2018) (** 7 **)**	Brazil	2004-2015		3.4	Reference center for diagnosis and treatment of adult leukemia patients in Pernambuco
**Kuan JW et al. (2018) (** 31 **)**	Malaysia	2011-2016	6.9 in 2016	0.8	Single but representative center in southern Sarawak
**Foulon et al. (2019) (** 10 **)**	France	2014	16.3		National Health Insurance database

In comparison with other studies performed outside France, the differences observed for the incidence estimates can be due to significant differences in the age distributions of the investigated populations and the geographical areas (e.g. Western vs several non-Western countries) (2, 6, 28). The differences may also be due to methodological issues (national extrapolation from regional registries, national exhaustive registries, single reference center, etc.) or differences in study periods. However, the difference between different geographical areas and/or ethnical sub-groups cannot be excluded to explain these incidence variations (7, 10, 31).

We observed variations in CML incidence across the different regions of the French territory but it should be taken with caution because these variations are based on crude incidence rates. To confirm it in our study, we should standardize the incidence rates in terms of age and gender. However, these geographic variations of CML incidence in France have also been described by Foulon et al. who studied the prevalence of CML in France using the SNDS (10). We agree with their assertion that this cannot be explained by a difference in the quality of data reporting across the regions because information on reimbursement of TKI, on which the algorithm mainly based, is collected in the same manner across the French territory. The role of unknown environmental factors in the incidence of CML cannot be excluded. However, data regarding environmental exposures are not available in the National Health Insurance databases. This should be confirmed by other studies across Europe.

With the increase in the incidence of CML and particularly in the elderly patients (peak of incidence at >75 years in our study), precautions should be taken by physicians in order to adapt their practices, follow-up and informing of CML patients (32). Elderly patients may be more sensitive to side effects and interactions with TKIs.

### Patterns of TKI Treatment and Healthcare Resources Consumption

This is the only recent study in France that examines patterns of TKI treatment among newly diagnosed CML patients in France between 2011 and 2014 (since the availability of second-generation TKI in 2006 and 2007 in France) (12, 33, 34). We observed that most of the patients were rarely or never hospitalized for CML on inclusion in the study or during the follow-up (15%). All the TKIs studied were delivered on an outpatient basis, on medical prescription and reimbursed by the health insurance, with one month’s treatment provided. Imatinib remains the most widely used drug, both in first-line and throughout the study period. However, we noticed an increase in the use of second-generation TKIs in the first-line setting overtime (18% were nilotinib and 4% dasatinib), which is consistent with previously published data, and this trend is more marked in young patients (27.8% and 7.5% of patients 18-39 years old were exposed respectively to nilotinib and dasatinib as first line treatment in this study) (33). Only approximately 14% of the patients changed TKI in their first year of treatment but there were discontinuations in 23% of the cases and almost 52% of interruptions. These results are consistent with studies that investigate treatment patterns with protein kinase inhibitors among patients with CML (35–38). Reasons for switching or discontinuation could be adverse effects or intolerance to TKI (38, 39). In fact, most of the patients who switched from one TKI to another (41%) did so during the first three months of treatment, before the first milestone evaluation according to international guidelines (12). In addition, most of these patients who switched TKI (69%) also interrupted treatment.

Our study reveals that patients with CML have other co-morbidities during management of their hematological pathology, 11% of patients had a previous history of cancer, which is high for a population with a median age of 60 years. Similar results have been previously reported in a population based-study including CML patients in France using regional data, with a highest proportion (18%) of CML patients previously treated for one or more other cancers before the CML occurrence, including lymphomas or other lymphoid diseases, prostate, breast, or digestive cancers (27, 40). The proportion of patients with comorbidities was underestimated in this study by using only those included in the CCI (hypertension or dyslipidemia or not complicated diabetes being not included). This is underlined by the proportion of patients exposed to antihypertensive agents or statins in the year before inclusion.

At the same time, in most instances CML patients often chronically receive numerous medications. For example, nearly half are treated with proton pump inhibitors, one-third receive antihypertensive drugs, benzodiazepines, opioid analgesics (a category including tramadol and codeine), or anti-platelet agents, one-quarter are exposed to statins, and 10% are exposed to antidepressants. This level of use confirms the results observed in other recent studies (41, 42). The concomitant use of TKI and other drug is of significant concern because of its impact on survival and therapy discontinuation in older adults with CML (42). There is also a risk of potential drug-drug interaction that reduces the effectiveness of TKIs (e.g., concomitant use of TKI and PPI reduces TKI absorption) (43).

### Identification of CML Cases in the SNDS

The use of the SNDS (a medico-administrative database) is the first and main strength of our study. It provides a representative and exhaustive sample of patients’ pathway of care in real-life conditions (98.8% of the French population); data are collected prospectively and are readily available with a unique identifier for each patient (which helps to avoid selection and attrition bias) (24, 26). Therefore, the SNDS is a powerful tool to conduct observational studies especially for rare diseases such as CML. Cancer identification algorithms have been developed based on SNDS data and validated on cancer registry data in various cancer sites (44, 45). Building such algorithms requires expertise in both the disease studied and the administrative databases used. Yet, there is still no validated algorithm for CML, which is a limitation of our study.

The algorithm used to identify incident CML patients was developed through collaborative work involving hematologists and pharmacoepidemiologists. A one-year period before inclusion (1/1/2010 -1/1/2011) was applied to ensure that patients were not previously treated for CML. Actually, we choose this one year period because a molecular relapse leading to a subsequent TKI re-treatment often occurs within 6 months after a first TKI discontinuation (46, 47). Patients treated with TKIs for other conditions were excluded to ensure that only patients treated for CML were selected. The fact that we identify CML patients based on TKI reimbursement appears robust given the current management recommendations (CML does not require hospital management in most cases) (14). The efficacy of these drugs has led to a systematic prescription in all newly diagnosed patients. In order to ensure the relevance of this algorithm, we have taken two different approaches to protect its robustness.

The first approach was to ask two clinicians, who were blinded of the ranking provided by the selection algorithm, to analyze the sequence of care of a randomly selected sample of patients in the cohort and to categorize patients as probable cases of CML or excluded CML. Overall, 90% of the subjects were correctly classified (true positives and true negatives). The second approach was to perform a sensitivity analysis to describe the demographics and management characteristics of incident CML patients from a tighter identification based on the assignment of an LTD for CML (with an LTD start date to confirm the onset of disease) and excluding diagnoses of acute leukemia (identified by the corresponding LTD codes). The total number of this cohort is almost half the number of patients in our study (hospital clinical data had already shown that less than half of patients treated for CML had LTD in this indication). Overall, this approach confirmed same socio-demographic characteristics (**Supplementary File 2** **Table S1**).

Our study also has limitations. The algorithm could not identify patients who had always received TKI in the setting of a clinical trial, in which case the TKI was provided by a sponsor and therefore not reimbursed by the National Health Insurance. In the EU Clinical Trials Register, there were 25 clinical trials on CML between 2004 and 2014 in France (48), which corresponds to an upper range of 628 prevalent patients that could have been missed by the algorithm (10). Among those patients, several discontinued the trials and were thus identifiable by the algorithm if treated by a TKI outside the trial. Therefore, the impact of these limitations on the estimation of the incidence of CML should be limited. Secondly, we were unable to validate CML patients’ identification with our algorithm using medical records because data from the French national health insurance databases for research are anonymous. This difficulty was partially overcome through internal validation. However, for the same reasons of anonymity, we were unable to perform individual matching with the registries. The algorithm could have overestimated the number of incidents CML patients. Non-CML patients who received TKI for others diseases besides the differential diagnoses already excluded by the algorithm may have led to this slight overestimation.

### Relevance and Limitations of the Use of SNDS in Measuring Drug Exposure

In pharmacoepidemiology, minimizing classification bias in the measurement of drug exposure is fundamental since the introduction of such bias can call into question the validity of the results obtained. In most studies, exposure can only be measured retrospectively and transversally with a risk of recall and non-response bias, while SNDS data collected continuously over time help avoiding those biases. Similarly, the qualitative aspect of drug consumption from these data sources may lack precision (dosage, duration of treatment, concomitant medications, etc.). Moreover, data on response rate to the TKI treatment and reasons for changes in treatment sequences were not available in the database.

## Conclusion

In conclusion, we built an algorithm to identify CML patients in healthcare administrative databases and described patterns of use of TKI treatment and healthcare consumption in incident CML patients treated with TKI in France between 2011 and 2014. In 2014, the estimated cumulative CML incidence rate was 1.37 per 100,000 inhabitants in France. The data analysis revealed that CML patients are mostly old, have other co-morbidities at the time of management of their hematological pathology, and at the same time, in most instances chronically receive numerous medications, but with little or no hospitalization for CML.

## Data Availability Statement

The raw data supporting the conclusions of this article will be made available by the authors, without undue reservation.

## Ethics Statement

The studies involving human participants were reviewed and approved by Institut des données de Santé. Written informed consent for participation was not required for this study in accordance with the national legislation and the institutional requirements.

## Author Contributions

MM and FD conceived the study. MP, CC, FH, and MG participated to study design, data management and statistical analysis plan. MP performed analysis and wrote the first draft. All the authors interpreted results and validated the manuscript’s content. MM is the scientific guarantor for the study. All authors contributed to the article and approved the submitted version.

## Funding

This study was performed in an academic setting in the context of the PhD of MP. The study was supported by a grant from the *Institut National du Cancer* (INCA) (Projets libres de recherche en sciences humaines et sociales, épidémiologie et santé publique) grant number SHSESP17-055.

**Figure:**
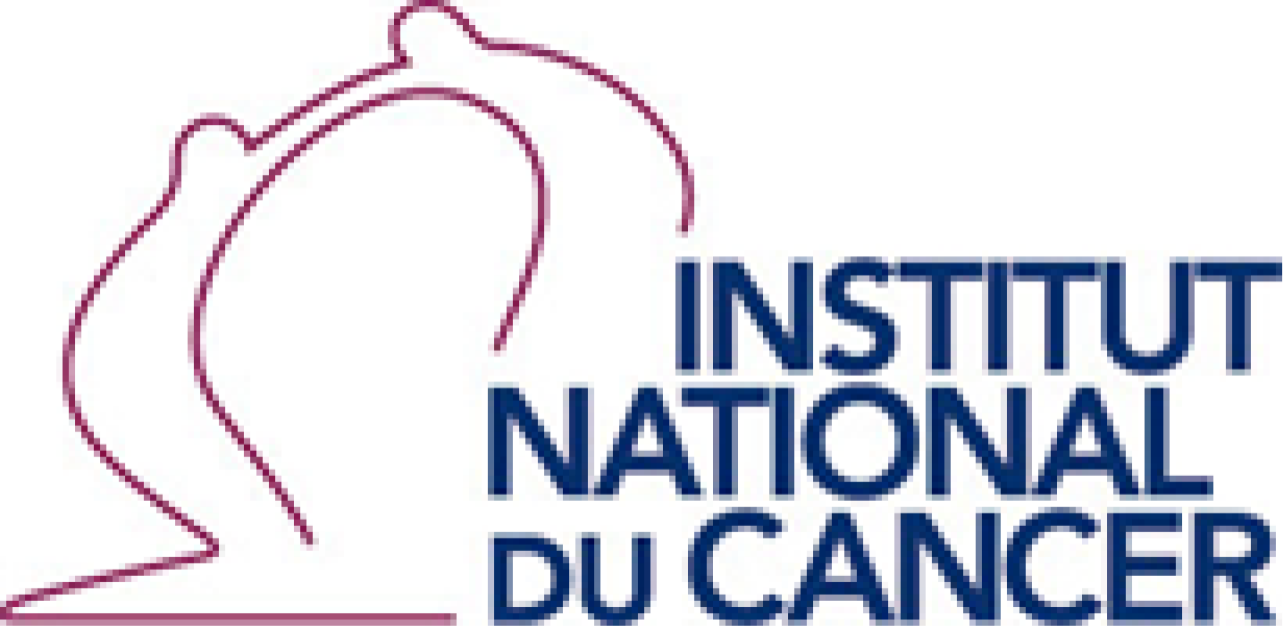


## Conflict of Interest

The authors declare that the research was conducted in the absence of any commercial or financial relationships that could be construed as a potential conflict of interest.

## Publisher’s Note

All claims expressed in this article are solely those of the authors and do not necessarily represent those of their affiliated organizations, or those of the publisher, the editors and the reviewers. Any product that may be evaluated in this article, or claim that may be made by its manufacturer, is not guaranteed or endorsed by the publisher.
