# Supplementary material for: Patterns of Tyrosine Kinase Inhibitor Utilization in Newly Treated Patients With Chronic Myeloid Leukemia: An Exhaustive Population-Based Study in France
Source: Front Oncol. 2021 Sep 30;11:675609. doi: 10.3389/fonc.2021.675609 (PMC8515137; doi:10.3389/fonc.2021.675609)
Supplement: Supplementary file 1 [file DataSheet_1.docx]

**SUPPLEMENTARY FILE 1**

- To eliminate DermatoFibroSarcoma (DFS): Patients treated with imatinib who do not have a C92 LTD code or C92 hospitalization code (main, related or combined diagnosis) and whose initial imatinib prescription is not from a hematologist and: The initial prescription for imatinib was prescribed by a dermatologist; (and/or) with a diagnosis of "malignant skin tumor" (ICD10 C44); (and/or) having an LTD "malignant skin tumor" (ICD10 C44).

- To eliminate Gastrointestinal Stromal Tumors (GIST): Patients treated with imatinib who do not have a C92 LTD code or C92 hospitalization code (main, related or combined diagnosis) and whose initial imatinib prescription is not from a hematologist and : Having a diagnostic code "malignant tumor of the digestive tract" (C15 to C21, C26.0, C26.0, C26.8, C26.8, C26.9) in PMSI (main, associated or related diagnosis) with the code of a diagnostic and/or therapeutic procedure compatible with biopsy and/or GIST removal (primary tumor or metastasis) for the same hospitalization; (and/or) with an LTD code "malignant tumor of the digestive tract" (C15 to C21, C26.0, C26.8, C26.9).

- To eliminate HyperEosinophilic Syndromes (HES): Patients treated with imatinib who do not have a C92 LTD or C92 hospitalization code (main diagnosis, related or associated) and: having a hospitalization code "hypereosinophilic syndrome" (D47.5) in main or related or associated diagnosis; (and/or) having an LTD code "hypereosinophilic syndrome" (D47).

- To eliminate Acute Lymphoblastic Leukemia (ALL Ph+): Patients with a primary hospitalization diagnosis of "acute lymphoblastic leukemia" (C91.0); (and/or) patients with a hospitalization-related diagnosis of "acute lymphoblastic leukemia" (C91.0) associated with case-mix coding of "chemotherapy" or "chemotherapy for acute leukemia", (and/or) patients with LTD "acute lymphoblastic leukemia" (C91).

- To eliminate Acute Myeloid Leukemia (AML): Patients with a diagnosis code "Acute Myeloid Leukemia" (C92.0, C92.4, C92.5, C92.6, and C92.8) in hospitalization (main, related, or associated diagnosis).

- To eliminate MyeloDysplastic Syndromes (MDS): Patients with a primary PMSI diagnosis code of “myelodysplastic syndrome” (D46.0 to D46.9), associated with a diagnosis of transfusion (Z513, Z5130, Z5131), and/or a diagnosis of chemotherapy (Z511), and/or patients with LTD “myelodysplastic syndrome” (D46).
