# Supplementary material for: Patterns of Tyrosine Kinase Inhibitor Utilization in Newly Treated Patients With Chronic Myeloid Leukemia: An Exhaustive Population-Based Study in France
Source: Front Oncol. 2021 Sep 30;11:675609. doi: 10.3389/fonc.2021.675609 (PMC8515137; doi:10.3389/fonc.2021.675609)
Supplement: Supplementary file 2 [file DataSheet_2.docx]

**SUPPLEMENTARY FILE 2:** Table **S**1: Description of population characteristics and comorbidities (in the 12 months preceding the index date) in incident "CML" subjects identified in the SNDS by the LTD code, 2011-2014, France

| Characteristics | N (%) |
| --- | --- |
| Number of subjects | 2812 |
| Age, years - median (interquartile range) | 58 (47-70) |
| Gender  Men  Women | 1,516 (53,91)  1,296 (46,09) |
| CMU-C *(complementary universal healthcare coverage)*  No  Yes | 2,598 (92,39)  170 (6,05) |
| Comorbidities | |
| Subjects with at least one LTD in the 12months preceding the index date | 2811 (99.96) |
| Subjects with at least one hospitalization in the 12months preceding the index date | 1,815 (65.54) |
| Charlson comorbidities index (CCI)  0  1  2  >2 | 2,112 (75.11)  152 (5.41)  415 (14.76)  133 (4.72) |
| Individual comorbidities according to CCI |  |
| Cancer (without CML) | 420 (15.00) |
| Peripheral vascular disease | 113 (4.02) |
| Chronic lung disease | 78 (2.77) |
| Myocardial infarction | 66 (2.35) |
| Heart failure | 65 (2.31) |
| Moderate to severe renal disease | 64 (2.28) |
| Mild liver disease | 45 (1.42) |
| Cerebrovascular pathology | 40 (1.42) |
| Connectivity | 24 (0.85) |
| Dementia | 23 (0.82) |
| Diabetes without complications | 19 (0.68) |
| Diabetes with complications | 17 (0.60) |
| Hemiplegia | 16 (0.57) |
| Metastatic pathology | 11 (0.39) |
| Ulcerative pathology | 10 (0.36) |
| Moderate to severe liver disease | 3 (0.11) |
| HIV-AIDS | 1 (0.04) |
